# Chirality dependence of spin current in spin pumping

**DOI:** 10.1038/s41467-022-32981-y

**Published:** 2022-09-05

**Authors:** Z. Q. Qiu

**Affiliations:** Department of Physics, University of California at Berkeley, Berkeley, CA 94720 USA

**Keywords:** Spintronics, Magnetic properties and materials

## Abstract

Chirality of magnons is an intrinsic degree of freedom that characterizes the handedness of spin precession around its equilibrium direction. This commentary summarizes recent progress on spin pumping by ferromagnetic resonance in magnetic heterostructures. In particular, the commentary highlights one fundamental issue in spin pumping: the chirality dependence of the spin current.

Chirality (or handedness) is an important concept that distinguishes a system from its mirror images. Magnons in ferromagnetic materials, the bosonic quasiparticles of collective spin excitations, have the unique character that only right-handed chirality exists in the spin precession around its equilibrium axis. Such right-handed spin precession could pump a pure spin current and create an electric voltage in neighboring layers, thus opening new opportunities for spintronics development^1^. Right-handed spin pumping has been successfully described by a phenomenological spin-damping term that the damped angular momentum of the spin precession converts into a spin current^2^. Nonetheless, the role of spin-precession chirality in spin pumping remains a fundamental issue. Specifically, it is unclear whether the right-handed and left-handed spin precessions pump the same spin current. Since nature selects only right-handed spin precession in ferromagnets, it is an experimental challenge to address this fundamental issue by investigating spin pumping from right-handed and left-handed spin precessions independently. Specifically, the experiment needs to answer if opposite chiralities of the spin precession should pump opposite spin polarizations of the spin current?

## Spin current from spin pumping

Akin to electric current, which is a flow of charge, spin current is a flow of spin that delivers angular momentum in space. Pure spin current, which consists of only the flow of spin without the flow of charge, mostly comes from the so-called spin pumping where the precession of a magnetic moment pumps a spin current into a neighboring layer due to the extra damping of $\triangle \alpha \vec{m}\times d\vec{m}/{dt}$, where $\vec{m}$ is the magnetic moment and $\alpha$ is the damping coefficient. Although the spin current described by the damping term is a time-dependent ac quantity^3–5^, its time average $\left\langle \vec{m}\times d\vec{m}/{dt}\right\rangle$ or DC component reflects the net change of the spin due to precession. This can be detected via the inverse spin Hall effect (ISHE), where the spin current is converted to a DC voltage^1^. Since the handedness of the spin procession is lost after time averaging, it is of fundamental interest to ask if a left-handed spin precession should pump the same spin current as a right-handed spin precession.

## Spin current in an antiferromagnetic insulator

**Fig. 1:**
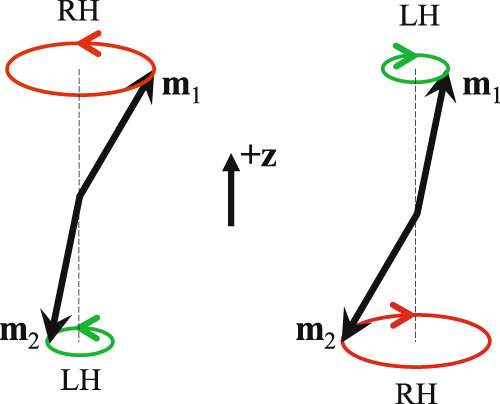
Schematic of the two eigenmodes of antiferromagnetic magnons in an easy-axis antiferromagnet. For both eigenmodes, the precessions of **m**_1_ and **m**_2_ have opposite chiralities around their equilibrium directions. Right-handed (left-handed) chirality is depicted in red (green).

In ferromagnets, only right-handed spin precession exists. In antiferromagnets (AFMs), however, the two antiferromagnetically coupled spins should precess around their equilibrium directions with opposite handedness (Fig. 1). As sketched in Fig. 1, the precessions of two magnetic sublattices (**m**_1_ and **m**_2_) in an AFM around their equilibrium directions always have opposite chirality in either of the two degenerate eigenmodes of magnons. For instance, the right-handed precession of **m**_1_ around the **+z** direction is accompanied by the left-handed precession of **m**_2_ around the **-z** direction. Recently, it was proposed that AFMs should be an effective spin current mediator because the spin currents carried by the precessions of two magnetic sublattices should be added together rather than be subtracted from each other^6^. This result raises the fundamental question of whether opposite chiralities of spin precessions should pump the same or opposite angular momentum of the spin current. In an AFM, it is impossible to separate the spin pumping signals from **m**_1_ and **m**_2_ precessions because of their coexistence, thus prohibiting the investigation of spin pumping from an isolated left-handed precession. Consequently, theorists have no choice but to accept the hypothesis that the damping term of $\vec{m}\times d\vec{m}/{dt}$ is valid for both right-handed and left-handed spin pumpings^6^. The angular momentums carried by **m**_1_ and **m**_2_ precessions are both described by the same damping term for spin current transmission in AFM insulators^7,8^. To address this fundamental issue, it is critical to realize an isolated left-handed spin precession and to study its spin pumping in an experiment.

## Chirality-dependent spin pumping

**Fig. 2:**
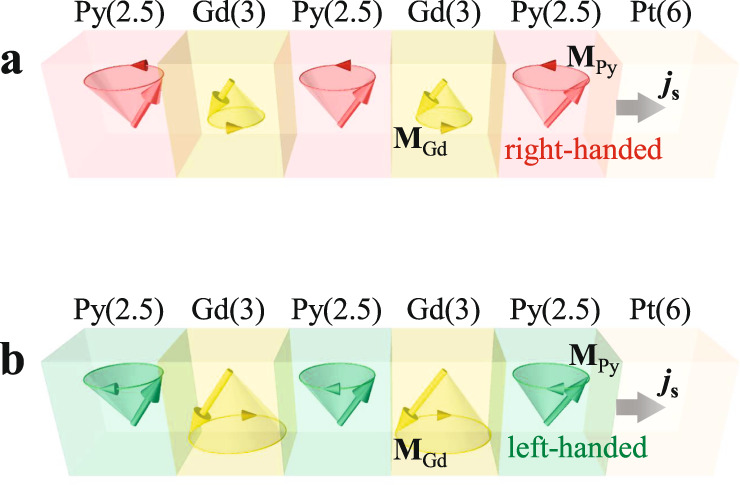
Schematic of chirality-dependent spin pumping from Py into Pt layers. The spin current injected into the Pt layer is generated by **a** right-handed **M**_Py_ precession in the Py-aligned phase (|**M**_Py_| > |**M**_Gd_|), and by **b** left-handed **M**_Py_ precession in the Gd-aligned phase (|**M**_Py_| < |**M**_Gd_|). The numbers in parentheses are thicknesses in the unit of nanometers.

One recent experiment designed a cleaver method in which spin pumping from Py into Pt layers by left-handed and right-handed Py magnetic precessions were realized independently^9^. In an artificial ferrimagnet of Py/Gd/Py/Gd/Py/Pt multilayer in which the Py and Gd are antiferromagnetically coupled, the net magnetization always precesses around a magnetic field with right-handed chirality, the same as the magnetization precession in a ferromagnet. Because the temperature-dependent Gd magnetization (**M**_Gd_) is stronger than the Py magnetization (**M**_Py_), there exists a transition from the Gd-aligned phase (|**M**_Py_| < |**M**_Gd_|) at low temperatures to the Py-aligned phase (|**M**_Py_| > |**M**_Gd_|) at high temperatures. Then the **M**_Py_ precession should have a right-handed chirality in the Py-aligned phase (Fig. 2a) and a left-handed chirality in the Gd-aligned phase (Fig. 2b). The left-handed **M**_Py_ precession does not exist in conventional ferromagnets because it is generated by its antiferromagnetic coupling to the right-handed **M**_Gd_ precession. Spin pumping by the right-handed and left-handed **M**_Py_ precessions could be realized at the Py/Pt interface and detected by the dc voltages via the ISHE. Therefore, spin pumping by isolated left-handed **M**_Py_ precession can be explored and compared to the result of right-handed **M**_Py_ precession without changing other parameters. As the main result of the experiment, Liu et al. demonstrated unambiguously that opposite chiralities pump opposite spin polarizations of the spin current. That is, the right-handed and left-handed **M**_Py_ precessions around the same axis produce opposite signs of the dc voltage via the ISHE. In addition, two different resonance modes were observed in this artificial ferrimagnet, corresponding to ferromagnetic antiferromagnetic excitations. Their work establishes the relationship between spin precession chirality and the electrical readout of spin pumping, demonstrating chirality as an independent degree of freedom and an information carrier in future spintronic devices.
